# Improving the Real-time Marine Forecasting of the Northern South China Sea by Assimilation of Glider-observed T/S Profiles

**DOI:** 10.1038/s41598-019-54241-8

**Published:** 2019-11-28

**Authors:** Shiqiu Peng, Yuhang Zhu, Zhijin Li, Yineng Li, Qiang Xie, Shijie Liu, Yeteng Luo, Yu Tian, Jiancheng Yu

**Affiliations:** 1 State Key Laboratory of Tropical Oceanography, South China Sea Institute of Oceanology, Chinese Academy of Sciences, Guangzhou, China; 2 Laboratory for Regional Oceanography and Numerical Modeling, Qingdao National Laboratory for Marine Science and Technology, Qingdao, China; 3 State Key Laboratory of Robotics, Shenyang Institute of Automation, Chinese Academy of Sciences, Shenyang, China; 4 Institute of Deep-sea Science and Engineering, Chinese Academy of Sciences, Sanya, China; 5 Joint Institute for Regional Earth System Science and Engineering, University of California, Los Angeles, CA USA; 6 Institution of South China Sea Ecology and Environmental Engineering, Chinese Academy of Sciences, Guangzhou, China; 7 University of Chinese Academy of Sciences, Beijing, 100049 China

**Keywords:** Hydrology, Physical oceanography

## Abstract

Prediction of marine conditions is notoriously challenging in the northern South China Sea (NSCS) due to inadequate observations in the region. The underwater gliders that were developed during the past decade may provide observing platforms that could produce required observations. During a field experiment, temperature/salinity (T/S) profiles from a set of underwater gliders were assimilated into a real-time marine forecasting system, along with the assimilation of climatological monthly mean Argo data to constrain the basin-wide model biases. The results show that, in addition to the reduction of the basin-wide model biases by the assimilation of the climatological monthly mean Argo data, the assimilation of glider-observed T/S profiles is efficient to reduce the local biases of the NSCS marine forecasting by as much as 28–31% (19–36%) in 24 h to 120 h forecasts for temperature (salinity) from sea surface to a depth of 1000 m. Our results imply that the real-time marine forecasting for the NSCS can largely benefit from a sustainable glider observing network of the NSCS in the future.

## Introduction

As the largest marginal sea in Northwest Pacific, the South China Sea (SCS) takes a key role in connecting the Pacific Ocean to the Indian Ocean. An accurate marine forecast in this region is highly required to guarantee the safety of navigation, fishery, engineering construction, and so on. The SCS enjoys a unique geographical condition. It is controlled by annually-reversing monsoon system and featured with many processes of regional ocean dynamics and physics such as basin-scale circulations^1,2^, strong western boundary currents^3^, overflows^4,5^, upwellings^6,7^, energetic mesoscale eddies^8–10^, internal tides^11,12^ and gravity waves^13^, thus its marine forecast remains a great challenge.

Numerical marine forecasting using an ocean circulation model relies on accurate initial conditions. Improving the accuracy of initial conditions is one of the most effective ways to reduce forecasting errors^14,15^. Data assimilation, which incorporates available observations into numerical models to reduce forecasting errors, is a common way at present to generate initial conditions of high accuracy for atmospheric or oceanic models^16–18^. There are different data assimilation algorithms, and currently the widely-used methods of data assimilation includes 3-dimensional/4-dimensional data assimilation (3DVAR/4DVAR)^19–23^ and the Ensemble Kalman Filter (EnKF)^24–26^. Each data assimilation approach has its own advantages and disadvantages, and the 3DVAR has still been adopted in most real time high-resolution (~ a few km) marine forecasting systems in the world due to relatively low cost^16,27,28^.

A variety of ocean surface observations have been provided by satellite-based remote sensing, but subsurface observations are very limited. Besides mooring buoys, Argo floats have been mainly used for subsurface measurements during the past couple of decades. They are generally located in the open ocean and are extremely sparse in coastal regions or marginal seas such as the SCS. Gliders work in a more controllable and flexible way than Argo floats and thus can be more suitable for coastal regions or marginal seas. In the past decade, gliders have been used increasingly in the world to measure temperature and salinity vertical profiles, especially in the coastal regions^29–31^.

In China, gliders have been under intensive development and become available in recent years. To our knowledge, however, no real time assimilation in a real-time marine forecasting system has been carried out yet in the SCS. Beginning in July 2018, a field experiment of observing the upper ocean using China designed and manufactured gliders took place in the northern SCS (NSCS). In this paper, we report the assimilation of T/S profile observations from those gliders into a real-time marine forecasting system and demonstrate their significant impacts on forecasting skills.

The rest of this paper is organized as follows. The next section gives a brief introduction of the real-time forecasting system of marine environment for the SCS and data assimilation method used, followed by a description of the field observation experiment and the obtained T/S profile observations in the NSCS in section 3. Skill assessments are presented in section 4. A summary is given in the final section.

## Field Observation Experiment and T/S Profile Observations

**Figure 1:**
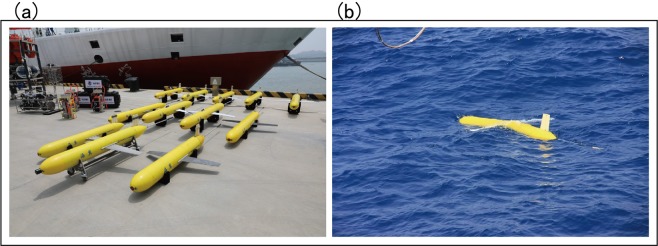
Pictures of Sea-Wing underwater gliders.

**Figure 2:**
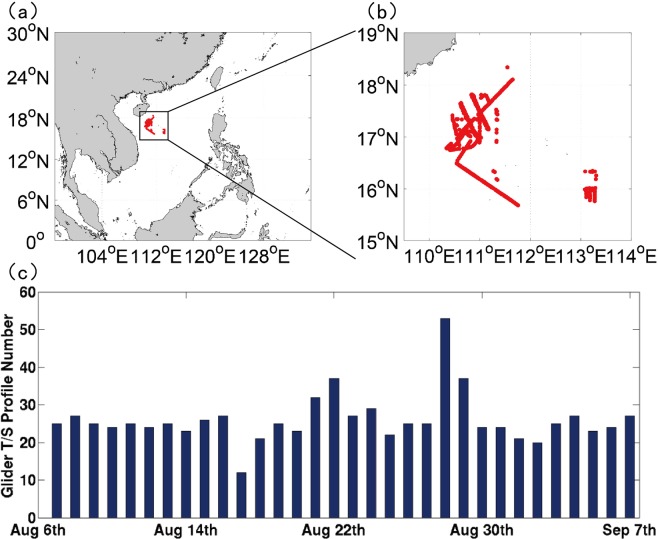
(**a**) Model domain of the NG-RFSSME and the region of the field experiment (denoted by the black square), (**b**) trajectories of the underwater gliders deployed in the field experiment and (**c**) the number of underwater gliders for each day from Aug 6 to Sep 7, 2018.

The field observation experiment in the NSCS started at August 4, 2018 and lasted for several months. Fourteen Sea-Wing underwater gliders (Fig. 1), developed by the State Key Laboratory of Robotics, Shenyang Institute of Automation, Chinese Academy of Sciences^32^, were deployed near the Xisha island to measure temperature and salinity profiles from the sea surface down a maximum depth of about 1000 m (Fig. 2). With a compact attitude-regulating unit and optimized steady gliding motion parameters, the Sea-Wing underwater glider was designed to save energy and increase gliding range^32,33^. Its performance has been comprehensively tested and assessed in field observation experiments during the past several years^34–36^.

The Sea-Wing underwater gliders dive and rise between the sea surface and the 1000-m depth with a V-like path; one cycle of diving and rising, which crosses a maximum horizontal distance of about 4 km and takes about 4 h, gains a T/S profile with vertical sampling resolution of 1 m. The trajectories of the underwater gliders during one month from Aug. 6 to Sept. 7 and the evolution of daily profile number are shown in Fig. 2, in which 819 T/S profiles were obtained.

### The real-time forecasting system and data assimilation method

The real-time forecasting system, called a New-Generation Real-time Forecasting System for the SCS Marine Environment (NG-RFSSME), is an updated version of the Experimental Platform of Marine Environment Forecasting (EPMEF)^37^ which was established in the State Key Laboratory of Tropical Oceanography (LTO), South China Sea Institute of Oceanology (SCSIO), in October 2010. It consists of three main components: an atmosphere model that employs the Weather Research and Forecasting (WRF) model, version 3.6^38^, an ocean circulation model that adopts the Princeton Ocean Model (POM), 2002 version^39,40^, and a sea wave model that uses the WAVEWATCH III (WWIII) model^41–43^. The WRF model, version 3.6, is a next-generation mesoscale numerical weather prediction system that was developed by the National Center for Atmospheric Research (NCAR) and the National Centers for Environmental Prediction (NCEP) to serve both operational forecasting and atmospheric research needs. A two-domain one-way-nested configuration is adopted for the WRF model with horizontal grid resolutions of 54 km and 18 km respectively and the inner domain covering the entire SCS and southern China (Fig. 2a), and there are 30 layers in the vertical for both domains. The initial and lateral boundary conditions of WRF model are the 6 hourly 1° × 1° output from the Global Forecast System (GFS) maintained by NCEP. The POM, developed in the Princeton University, is a three-dimensional (3D) primitive-equation ocean model embedded with a second-moment turbulence closure model (the Mellor–Yamada level 2.5 scheme)^44^, it covers the entire SCS domain (Fig. 2a) with a horizontal resolution of 1/15 and 40 layers in the vertical and is two-way fully coupled to the inner-domain WRF model with a coupler OASIS3 (Ocean Atmosphere Sea Ice Soil III)^45^ regarding to the heat and momentum flux exchanges between the ocean and the atmosphere; the climatologically monthly mean SODA (Simple Ocean Data Assimilation)^46,47^ data are used for the open boundary conditions of the temperature/salinity/currents, and the OTPS (Oregon State University Tidal Prediction Software)^48^ provides tidal levels and currents of 13 main tidal constituents at open boundaries. The WWIII model, which was developed at NOAA/NCEP, is one-way coupled to the inner-domain of WRF model from which the 10-m-height winds are used as the dynamical forcing of the wave model.

Data assimilation modules are constructed separately for the atmospheric component and the oceanic component of the NG-RFSSME: in addition to the built-in WRF-3DVAR data assimilation system, a “scale-selective data assimilation” (SSDA) scheme^49–52^ is incorporated in the WRF model, while a multi-scale 3DVAR (MS-3DVAR) data assimilation scheme^27,53–56^ is applied to the POM model. In the SSDA scheme, a low pass filter is employed to perform scale separation on the wind fields from both GFS (6 hourly 1° × 1°) and WRF outputs, and then the large-scale component of GFS wind field is assimilated to adjust the large-scale component of the WRF wind field using 3DVAR method; the adjusted large-scale component of the WRF wind field is recombined with the unchanged small scale component of the WRF wind field to be a new wind field for the initial conditions at each forecast cycle. The MS-3DVAR takes into account the large- and small-scale information represented by different sampling density of different observation types, e.g., the sparse T/S profiles and ship-track sea surface temperature (SST) and the dense satellite-derived sea surface height anomaly (SSHA) and SST, and thus can effectively suppress or remove the spurious adjustments caused by the scale mismatch among different observations. Technically, the cost function to be minimized is divided into two parts:

1 $${J}_{L}(\delta {{\boldsymbol{x}}}_{L})=\frac{1}{2}\delta {{\boldsymbol{x}}}_{L}^{T}{{\bf{B}}}_{L}^{-1}\delta {{\boldsymbol{x}}}_{L}+\frac{1}{2}{({\bf{H}}\delta {{\boldsymbol{x}}}_{L}-\delta {{\boldsymbol{y}}}_{L})}^{T}{({\bf{R}}+{\bf{H}}{{\bf{B}}}_{S}{{\bf{H}}}^{T})}^{-1}({\bf{H}}\delta {{\boldsymbol{x}}}_{L}-\delta {{\boldsymbol{y}}}_{L}),$$

2 $${J}_{S}(\delta {{\boldsymbol{x}}}_{S})=\frac{1}{2}\delta {{\boldsymbol{x}}}_{S}^{T}{{\bf{B}}}_{S}^{-1}\delta {{\boldsymbol{x}}}_{S}+\frac{1}{2}{({\bf{H}}\delta {{\boldsymbol{x}}}_{S}-\delta {{\boldsymbol{y}}}_{S})}^{T}{({\bf{R}}+{\bf{H}}{{\bf{B}}}_{L}{{\bf{H}}}^{T})}^{-1}({\bf{H}}\delta {{\boldsymbol{x}}}_{S}-\delta {{\boldsymbol{y}}}_{S}),$$

in which the subscripts *L* and *S* represent large- and small-scale, respectively, and the superscript *T* represents the transpose operator. ${\rm{\delta }}{\boldsymbol{x}}={\boldsymbol{x}}-{{\boldsymbol{x}}}^{b}$ is the increment of optimal values of the model variable vector ***x*** relative to their background values ***x***^b^, while ${\rm{\delta }}{\boldsymbol{y}}={\boldsymbol{y}}-{\bf{H}}{{\boldsymbol{x}}}^{b}$ represents the deviation of observations ***y*** from their corresponding model output **H*x***^*b*^. **H**, **B** and **R** are the Jacobian matrix of the nonlinearly observational operator, the background error covariance matrix and the observational error covariance matrix, respectively. Please be aware that the large-scale cost function (*J*_*L*_) (1) includes small-scale background error covariance matrix **B**_*S*_ as the representativeness error (${\bf{H}}{{\bf{B}}}_{S}{{\bf{H}}}^{T}$) of observations, and vice versa; this dealing could help to reduce or eliminate the representativeness errors of observations, and thus improve the performance of the data assimilation with different sampling density of various observation types. Due to the huge dimensions of **B**_*L*_ (or **B**_*S*_), the construction of **B**_*L*_ (or **B**_*S*_) is a very tough work; to reduce the dimensions for a easy construction, here **B**_*L*_ (or **B**_*S*_) is decomposed into the standard deviation matrices and horizontal or vertical correlation matrices. Then the NMC method^57^ is used to estimate the standard deviation matrices and the Kronecker product^58^ is employed to decompose the 3-D matrices of horizontal and vertical correlation into 1-D matrices in both scales. In practice, the scale separation is achieved by performing the data assimilation procedure (including the calculation of matrix B) on coarse and fine model grids sequentially, in which the fine grid is the same as the original grid of the ocean model, while the coarse grid is 3 times the original grid. The large-scale data assimilation is first conducted to generate the large-scale increment $\delta {{\boldsymbol{x}}}_{L}$, which is then interpolated and added to the small scale background field ${{\boldsymbol{x}}}_{S}^{b}$ for the small-scale data assimilation. Therefore, $\delta {{\boldsymbol{y}}}_{S}$ is not equal to $\delta {{\boldsymbol{y}}}_{L}$ due to the difference of background fields between the small and large scales. Readers may refer to Li *et al*.^27^ or Peng *et al*.^56^ for details.

### The effect of assimilating glider observations in improving the marine forecasting

**Figure 3:**
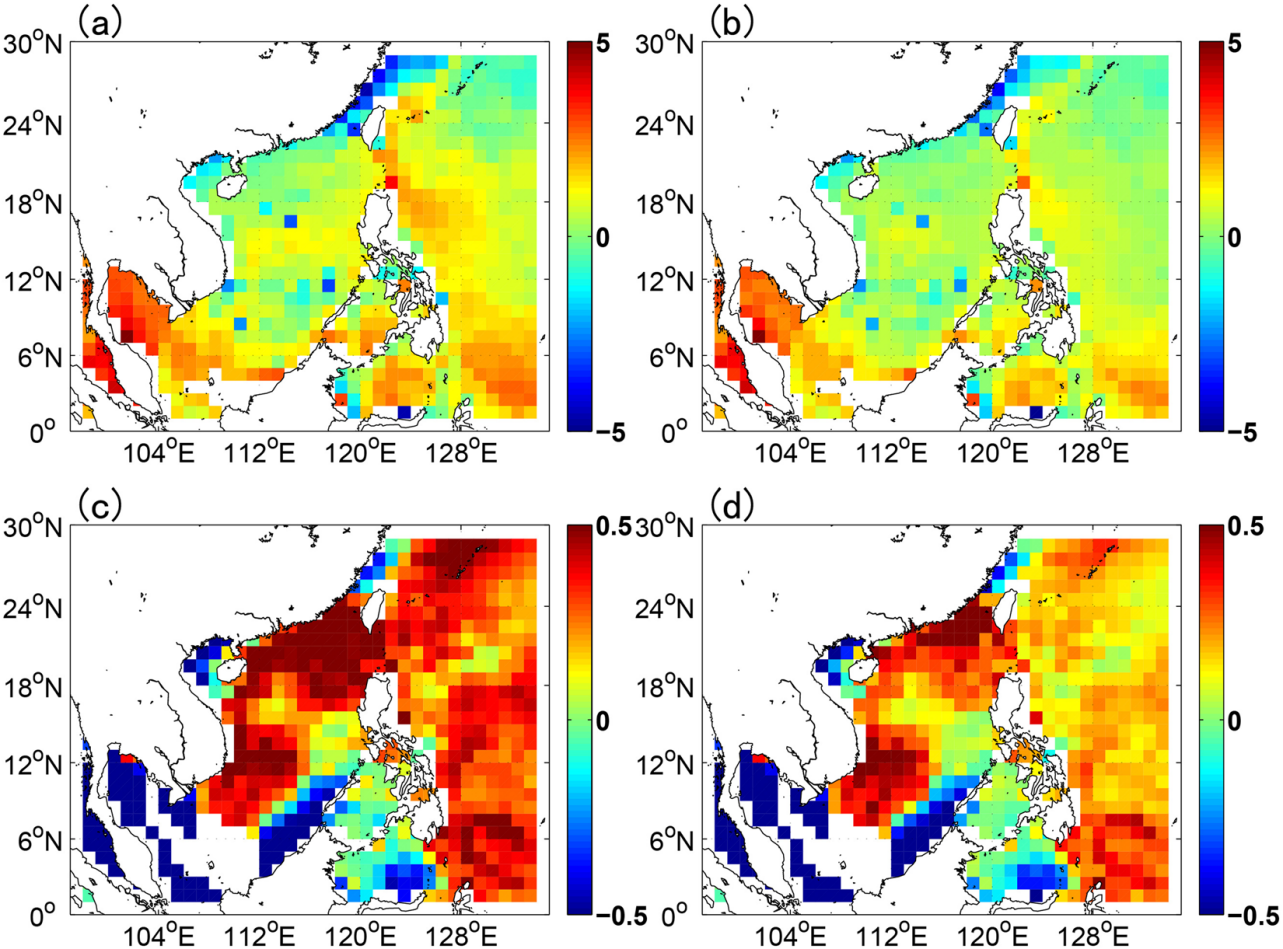
The biases of SST (unit: °C) (**a**,**b**) and SSS (unit: psu) (**c**,**d**) from model output before (**a**,**c**) and after (**b**,**d**) the assimilation of the climatological monthly mean Argo data at 0000 UTC Aug 7.

Due to the heavily localizing feature of glider observations (Fig. 2) and the sparseness of the real-time Argo profiles in the NSCS, the climatological monthly mean Argo data, generated from the three-dimensional grid dataset (2004–2015) of Argo provided by China Argo Real-time Data Center^59^, were assimilated to constrain the basin wide biases of the model in the NSCS at 0000 UTC of each day before the assimilation of glider-observed T/S profiles through the MS-3DVAR scheme. Figure 3 shows the biases of SST and sea surface salinity (SSS) from the model output before and after the assimilation of the climatological monthly mean Argo data; it is found that the biases of both SST and SSS were reduced obviously after the assimilation of the Argo data. Then the real-time T/S profiles obtained by gliders, which were subject to a quality control and then interpolated into the standard vertical layers, were assimilated into the POM of NG-RFSSME every 6 hours (i.e., at 0000 UTC, 0600 UTC, 1200 UTC and 1800 UTC) using the MS-3DVAR scheme, which is denoted as DA_CLIM-ARGO + GLIDER. As a preliminary result, a period of one month from Aug. 7 to Sept. 8 was selected for the assessment of the effect of assimilating the glider observations. For comparison, two paralleling experiments, i.e., one without any data assimilation and one with only climatological monthly mean Argo data assimilated, were performed for the same period, which are denoted as NODA and DA_CLIM-ARGO, respectively. A 5-day forecasting was made 4 times each day at 0000 UTC, 0600 UTC, 1200 UTC and 1800 UTC for all the experiments, and the forecasting results were validated against the “independent” glider observations (i.e., these observations had not been assimilated into the model by the validation time). For a more accurate evaluation on the role of glider observations in improving the marine forecasting skills, the satellite-derived SST/SSH and the real-time Argo T/S profiles were not assimilated in these experiments.

**Figure 4:**
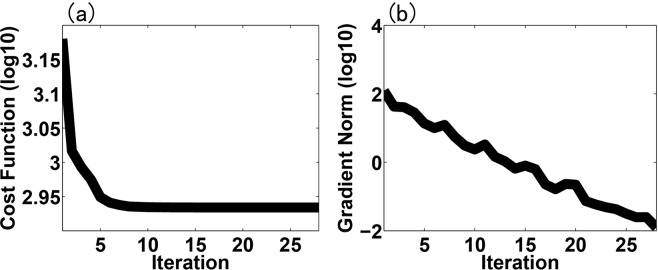
The evolution of (**a**) the small scale cost function (*J*_*S*_) value and (**b**) its gradient norm in logarithmic scale with iteration number of the minimum procedure during the data assimilation cycle on 0000 UTC Aug 7.

**Figure 5:**
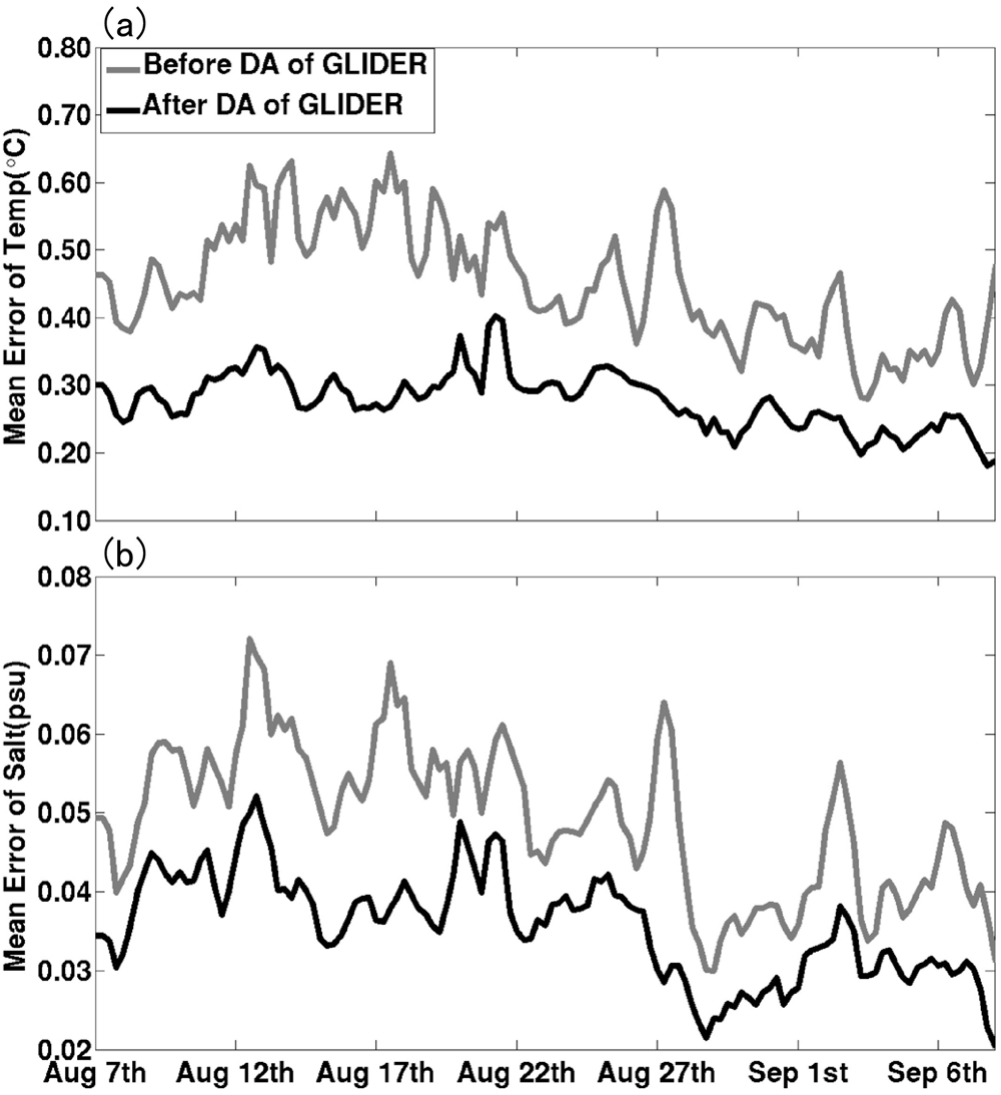
The vertically-integrated mean biases of (**a**) temperature and (**b**) salinity before (grey line) and after (black line) data assimilation of glider T/S profiles at each cycle validated against the glider observations during the one-month period of 7 Aug. to 8 Sept. 2018.

**Figure 6:**
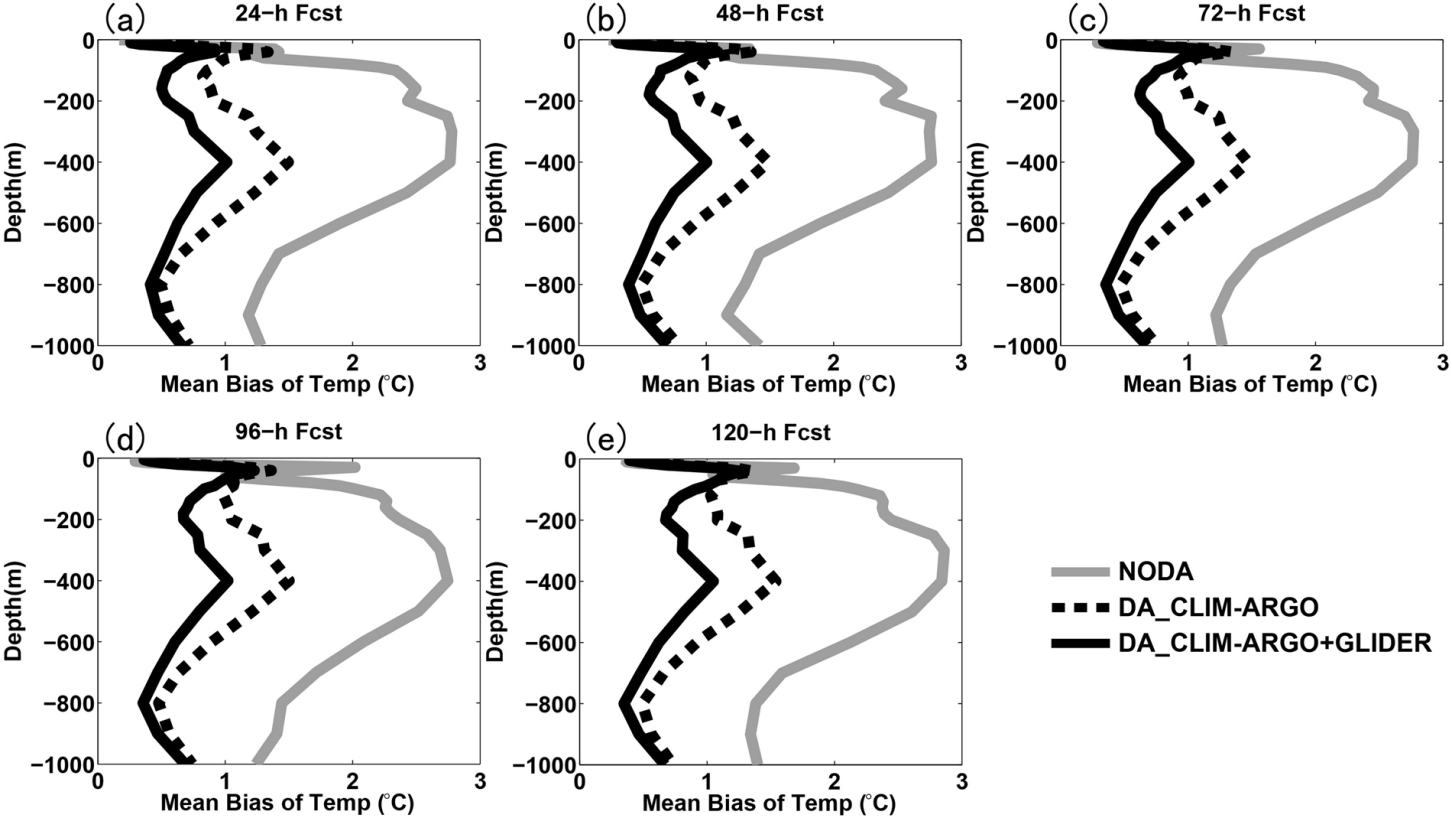
The mean biases of vertical temperature profiles for (**a**) 24-h, (**b**) 48-h, (**c**) 72-h, (**d**) 96-h and (**e**) 120-h forecasts from NODA (grey solid line), DA_CLIM-ARGO (black dotted line) and DA_CLIM-ARGO + GLIDER (black solid line) validated against the “independent” glider observations.

**Figure 7:**
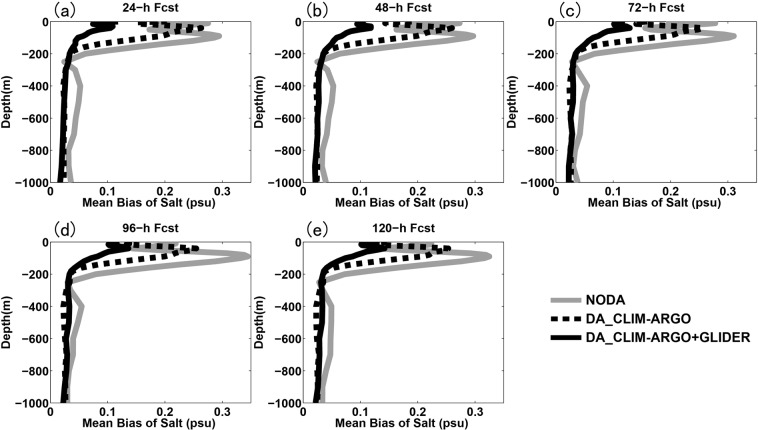
The same as Fig. 6, except for salinity.

Figure 4 shows the evolution of the small scale cost function (*J*_*S*_) value and its gradient norm in logarithmic scale with iteration number of the minimum procedure during one data assimilation cycle on 0000 UTC Aug. 7. It can be seen that the gradient norm of the cost function reduced to near zero at 10^th^ iteration with cost function value reducing to about a half of the original value, implying that the MS-3DVAR system works well in the assimilation of glider observations. The vertically-integrated mean biases of temperature and salinity fields before and after data assimilation of glider T/S profiles at each cycle validated against the glider observations during the whole month are shown in Fig. 5, which are found to reduce from about 0.45 °C and 0.05 psu to about 0.28 °C and 0.04 psu that are close to the preset observational errors, respectively, further confirming the well digestion of glider observations by the MS-3DVAR system. Figures 6 and 7 give the mean biases of vertical T/S profiles for 24 h, 48 h, 72 h, 96 h and 120 h forecasts from NODA, DA_CLIM-ARGO and DA_CLIM-ARGO + GLIDER validated against the “independent” glider observations. The results demonstrate that, assimilating only the climatological monthly mean Argo data (DA_CLIM-ARGO) helped to reduce the model biases which could be basin-wide, and the assimilation of the real-time glider-observed T/S profiles was able to achieve an additional bias reduction of about 28–31% (19–36%) for the 24~120 h forecasts of temperature (salinity) from sea surface to a depth of 1000 m (Table 1). Therefore, the glider observations could be very useful in improving the real time marine forecasting skills in the NSCS, especially for shorter forecasting periods, while it is also much beneficial and necessary to assimilate the climatological monthly mean Argo data for constraining the basin-wide model biases before assimilating these much localized glider observations.

**Table 1:** The vertically-integrated mean bias reduction of temperature and salinity for different forecast periods from DA_CLIM-ARGO + GLIDER and DA_CLIM-ARGO compared to NODA, and DA_CLIM-ARGO + GLIDER compared to DA_CLIM-ARGO.

Forecast Period		24 h	48 h	72 h	96 h	120 h	Mean
DA_CLIM-ARGO + GLIDER VS NODA	Temp	67.3%	66.6%	66.4%	65.7%	65.7%	66.3%
	Salt	58.3%	53.9%	51.2%	49.5%	48.7%	52.3%
DA_CLIM-ARGO VS NODA	Temp	52.4%	52.0%	52.3%	52.0%	51.8%	52.1%
	Salt	34.4%	34.1%	33.9%	35.5%	36.0%	34.8%
DA_CLIM-ARGO + GLIDER VS DA_CLIM-ARGO	Temp	31.3%	30.6%	29.6%	28.5%	28.8%	29.8%
	Salt	36.5%	30.1%	26.1%	21.7%	19.9%	26.9%

## Summary

In this study, the role of assimilating glider observations in NSCS from a field observation experiment into a sophisticated marine forecasting system is evaluated. The Sea-Wing underwater gliders, which were designed and manufactured by the State Key Laboratory of Robotics, Shenyang Institute of Automation, Chinese Academy of Sciences, were deployed in the field observation experiment and 819 T/S profiles from the sea surface down a maximum depth of about 1000 m obtained during one month of Aug. 6 to Sept. 7 were assimilated using a multi-scale 3-D variation data assimilation method. The climatological monthly mean Argo data were assimilated to constrain the basin-wide model biases of the NSCS before the assimilation of the glider-observed T/S profiles due to the heavily localizing feature of glider observations and the sparseness of the real-time Argo profiles in the NSCS. The results demonstrate that the assimilation of glider-observed T/S profiles was able to improve the forecasting of the marine environment in the NSCS significantly, with a bias reduction of about 28–31% (19–36%) in one-day to five-day forecasting for temperature (salinity) in addition to the bias reduction by the assimilation of climatological monthly mean Argo data. Our results demonstrate that the glider observations are very useful in improving the real time marine forecasting skills in the NSCS, especially for shorter forecasting periods, while it is also much beneficial and necessary to assimilate the climatological monthly mean Argo data for constraining the basin-wide model biases before assimilating glider observations under the situation of rare real-time Argo observations in the NSCS.

Our results imply that a sustainable glider observing network of the NSCS is necessary and valuable to improve the real-time marine forecasting of the NSCS in the future. However, before its operational application in the real-time marine forecasts for the NSCS, some issues still need to be clarified: 1) what is the optimal sampling density of glider observations in both space and time for the data assimilation to improve the marine forecasting in the NSCS? 2) What is the combining effect of assimilating simultaneously glider observations and other types of observations such as the satellite-observed SSH and SST? And 3) besides the temperature and salinity, does the assimilation of glider-observed T/S profiles has any improvement in the forecasting of ocean currents? These issues will be investigated in our future work along with more field observation experiments in the NSCS.

## Data Availability

The SODA dataset is provided by NCAR, the GFS dataset is provided by NCEP, the tidal constituents used for the lateral boundary of POM is provided by the OTPS developed by Oregon State University, the climatological monthly mean Argo is provided by China Argo Real-time Data Center, and the underwater glider observed T/S profiles are provided by the State Key Laboratory of Robotics, Shenyang Institute of Automation, Chinese Academy of Sciences, and are available upon cooperation with the Shenyang Institute of Automation.
